# Exploring the multifaceted role of direct interaction between cancer cells and fibroblasts in cancer progression

**DOI:** 10.3389/fmolb.2024.1379971

**Published:** 2024-05-28

**Authors:** Nilu Dhungel, Ana-Maria Dragoi

**Affiliations:** ^1^ Department of Molecular and Cellular Physiology, LSUHSC, Shreveport, LA, United States; ^2^ Feist-Weiller Cancer Center, INLET Core, LSUHSC, Shreveport, LA, United States

**Keywords:** TME, cancer-associated fibroblasts, chemotherapy resistance, EMT-epithelial to mesenchymal transformation, direct cell-cell communication

## Abstract

The interaction between the tumor microenvironment (TME) and the cancer cells is a complex and mutually beneficial system that leads to rapid cancer cells proliferation, metastasis, and resistance to therapy. It is now recognized that cancer cells are not isolated, and tumor progression is governed among others, by many components of the TME. The reciprocal cross-talk between cancer cells and their microenvironment can be indirect through the secretion of extracellular matrix (ECM) proteins and paracrine signaling through exosomes, cytokines, and growth factors, or direct by cell-to-cell contact mediated by cell surface receptors and adhesion molecules. Among TME components, cancer-associated fibroblasts (CAFs) are of unique interest. As one of the most abundant components of the TME, CAFs play key roles in the reorganization of the extracellular matrix, facilitating metastasis and chemotherapy evasion. Both direct and indirect roles have been described for CAFs in modulating tumor progression. In this review, we focus on recent advances in understanding the role of direct contact between cancer cells and cancer-associated fibroblasts (CAFs) in driving tumor development and metastasis. We also summarize recent findings on the role of direct contact between cancer cells and CAFs in chemotherapy resistance.

## Introduction

The “seed and soil” hypothesis, introduced by Stephen Paget in 1889, was the first concept to suggest that the TME is a crucial factor in controlling the growth and metastasis of cancer cells (Paget, 1989). His hypothesis laid the foundation for understanding how a specific environment (“soil”) influences the behavior of the cancer cells (“seed”) (Langley and Fidler, 2011). Although this concept is more than a century old, the critical involvement of supporting stroma in cancer progression is just beginning to be fully understood as novel findings reshape this complex interaction. Therefore, Paget’s original model has been revised by new research demonstrating that both the tumor and the TME display phenotypic plasticity and that intratumoral heterogeneity and continuous adaptation to the surroundings are critical for both cancer cells and TME during cancer evolution (Santiago-Gómez et al., 2023).

Solid tumors are surrounded by a TME made up of cellular components including fibroblasts, immune cells, endothelial cells, dendritic cells, mesenchymal stem cells, and non-cellular components including collagens, proteoglycans, fibronectin, laminins, and elastin among others (Anderson and Simon, 2020). The TME plays a critical role in cancer growth, metastatic invasion, and response to therapy due to the continuous cross-talk with the tumor (Pickup et al., 2014; Henke et al., 2019; Hinshaw and Shevde, 2019; Sung et al., 2021).

Within the TME, cancer-associated fibroblasts (CAFs) constitute the majority of the cellular component (Tlsty and Hein, 2001). CAFs promote stemness, angiogenesis, immune evasion, and extracellular matrix remodeling, all critical factors in cancer development and resistance to therapy (LeBleu and Kalluri, 2018; Liu et al., 2019; Sahai et al., 2020). Cancer cells themselves induce and maintain CAFs in an active state (Gupta and Massague, 2004; Nair et al., 2017; Valenti et al., 2017; Wang et al., 2022), thus promoting a reciprocally favorable link between the 2 cell types. The presence of activated CAFs has been associated with cancer recurrence and the worst outcome (Calon et al., 2015; Liu L. et al., 2016; Cords et al., 2024). Even more, significant roles for CAFs have been recently described in regulating tumor dormancy (Wu et al., 2022; Cheng et al., 2023).

With breakthrough technologies and the emergence of the “omics” era, we are now beginning to understand the tumor evolution in its entirety, as governed by the “soil” with its cellular and acellular components. The current chemotherapy and immunotherapy largely target the cancer cells, not the TME or the CAFs. However, increasing evidence indicates that the TME and CAFs regulate cancer cells metastasis and promote chemotherapy resistance. Therefore, establishing new therapies relies on a more profound understanding of the role of CAFs in tumor progression. Disrupting the interaction and communication between CAFs and cancer cells could be a turning point in therapy resistance.

Previous reviews have extensively covered mechanisms of CAFs activation and indirect regulation of tumor cells as well as the heterogeneity of the CAFs phenotypes in individual cancers (Eble and Niland, 2019; Fiori et al., 2019; Barrett and Puré, 2020; Biffi and Tuveson, 2021; Chhabra and Weeraratna, 2023; Wieder, 2023). In this review, we focus on the specific role of direct contact between CAFs and cancer cells in cancer progression. We will briefly examine different mechanisms of communication between the 2 cell types and explore in detail novel findings related to the direct cell-to-cell contact between the tumor and CAFs and its impact on tumor progression. We will also discuss the therapeutic implications of the direct contact between the tumor cells and CAFs.

## Cancer-associated fibroblasts

Since their first description as a distinct cell type by Rudolf Virchow in 1858, significant advancements have been made in understanding the roles and functions of fibroblasts (Plikus et al., 2021). As the most common cells in the connective tissue, fibroblasts play integral roles in extracellular matrix deposition and remodeling, secretion of signaling factors (cytokines and growth factors), generation of mechanical forces through contractility, and tissue metabolism (Plikus et al., 2021). Importantly, there is a remarkable degree of heterogeneity in fibroblasts based on the anatomical origin which ultimately dictates shared and specific functions of the fibroblasts (Muhl et al., 2020). Under physiological conditions, fibroblasts are in a dormant state and become activated during wound healing or fibrosis (Kalluri, 2016). In normal wound healing, activated fibroblasts are eliminated through programmed cell death once the wound is healed (Eming et al., 2014) whereas the CAFs persist, resembling a non-healing wound (Chhabra and Weeraratna, 2023).

CAFs can also originate from various sources including normal fibroblasts, epithelial cells, endothelial cells, bone marrow-derived mesenchymal stem cells (MSCs), hematopoietic stem cells, cancer stem cells (CSCs), adipocytes, pericytes, and stellate cells (Kobayashi et al., 2019; Ping et al., 2021; Kotsiliti, 2022; Wieder, 2023) via several processes such as activation, recruitment, differentiation, and transdifferentiation (Ansardamavandi and Tafazzoli-Shadpour, 2021; Chhabra and Weeraratna, 2023). This diversity in origin also contributes to the heterogeneity of CAFs (Ping et al., 2021; Chhabra and Weeraratna, 2023). Due to their heterogeneity, identifying specific markers for CAFs proved to be a challenge. Therefore, cell populations in the tumor that follow several criteria are defined as CAFs: 1) lack of original tumor cell mutations; 2) presence of mesenchymal markers, such as vimentin (VIM), alpha-smooth muscle actin (α-SMA), and fibroblast activating protein (FAP); 3) spindle morphology. Nonetheless, it is believed that a majority of the CAFs are formed from the activation of normal fibroblasts of the neighboring tumor site through various stimuli and activating signals (Arina et al., 2016; Öhlund et al., 2017).

**FIGURE 1:**
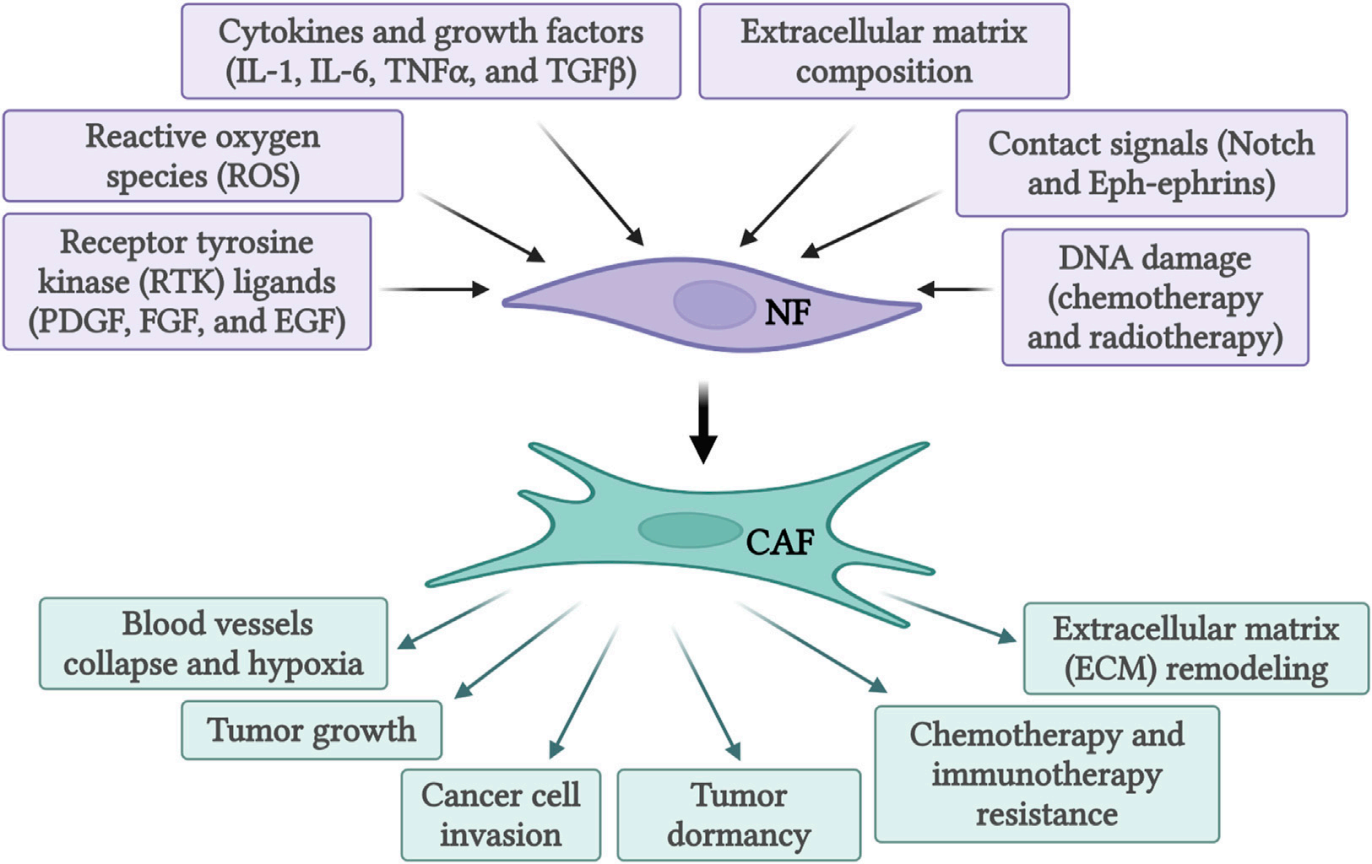
Activation of normal fibroblasts (NF) into cancer-associated fibroblasts (CAF) by various activating signals and subsequent functions of activated CAFs. Created with BioRender.com.

The recruitment and activation of CAFs are a result of cytokines and growth factors secretion by the tumor cells themselves and infiltrating immune cells (Figure 1). Among these growth factors, transforming growth factor beta (TGF-β) is particularly important (Casey et al., 2008; Kuzet and Gaggioli, 2016; Huang et al., 2021). This is due to its extensive role in cellular signaling and activation of genes promoting epithelial-to-mesenchymal transition (EMT), cell migration and invasion, and resistance to therapy (Massague and Gomis, 2006; Massague, 2008; Massague, 2012). Similarly, signals arising from the tumor site, including reactive oxygen species and ECM stiffness can promote CAFs conversion (Chhabra and Weeraratna, 2023). Activated CAFs fall into several categories defined by their function, specific markers, and transcription factors specifying the CAFs subtypes. The extensive heterogeneity in CAFs phenotypes in diverse cancers has been comprehensively reviewed (Biffi and Tuveson, 2021; Plikus et al., 2021; Chhabra and Weeraratna, 2023; Melchionna et al., 2023). As our understanding of CAFs heterogeneity increased, different studies identified specific subsets of CAFs in various tissues (see Table 1). In pancreatic cancer, for example, prevalent subtypes are inflammatory CAFs, defined by the expression of IL6, IL11, IL8, CXCL12, and other cytokines (Öhlund et al., 2017; Bernard et al., 2019), and myofibroblastic CAFs, defined by the expression of α-SMA, TGF-β, FAP, periostin (POSTN), and podoplanin (PDPN) (Öhlund et al., 2017; Bernard et al., 2019). Nonetheless, additional markers for inflammatory and myofibroblastic CAFs have been identified in both pancreatic and breast cancers (Fearon, 2014; Kawase et al., 2015; Öhlund et al., 2017; Djurec et al., 2018; Biffi et al., 2019; Elyada et al., 2019; Miyai et al., 2020). Based on FAP, α-SMA, CD29 and MCAM expression additional subsets of CAFs were identified in breast cancer (Costa et al., 2018; Kieffer et al., 2020; Pelon et al., 2020). Antigen-presenting CAFs were also identified in pancreatic and breast cancer (Djurec et al., 2018; Elyada et al., 2019). Multiple lung cancer studies found diverse clusters of CAFs as well (Lambrechts et al., 2018; Hu et al., 2021; Grout et al., 2022). For example, a lung cancer study identified different CAFs subsets in high-stage vs*.* low-stage tumors and associated with T cell exclusion or T cell enrichment (Grout et al., 2022). Another lung cancer study categorized CAFs into three different subsets based on the expression of HGH, FGF7, and phospho-SMAD2. These subsets were associated with different clinical outcomes (Hu et al., 2021). Recently, glycolytic CAFs have been described in soft tissue sarcomas (Broz et al., 2024).

**TABLE 1:** CAFs subtypes and markers.

Cancer type	CAFs markers by subtype	References
Pancreatic cancer	αSMA, VIM, CTGF, COL1A1, COL5A1, COL6A1, FAP (myofibroblastic CAFs)	Ohlund et al. (2017), Fearon. (2014), Miyai et al. (2020), Kawase et al. (2015)
	IL6, IL1, IL11, LIF, CXCL12 (inflammatory CAFs)	Ohlund et al. (2017), Elyada et al. (2019), Biffi et al. (2019)
	PDGFRα, SAA3, HLA-DR, CD74, SLPI (antigen presenting CAFs)	Djurec et al. (2018), Elyada et al. (2019)
Breast cancer	FAP^high^, αSMA^high^, MCAM^low^ (CAF subset 1) FAP^negative^, αSMA^negative^, CD29^low^ (CAF subset 2)FAP^negative^, αSMA^negative^, CD29^positive^ (CAF subset 3)FAP^negative^, αSMA^high^, CD29^high^, MCAM^high^ (CAF subset 4)	Costa et al. (2018), Kieffer et al. (2020), Pelon et al. (2020)
Lung cancer	COL10A1, TGF-β (Cluster 1)COL4A1, αSMA (Cluster 2)PLA2G2A (Cluster 4)MMP3 (Cluster 5)CCL2 (Cluster 7)	Lambrechts et al. (2018)
	HGF^high^, FGF7^high/low^, pSMAD2^low^ (CAF subset I)HGF^low^, FGF7^high^, pSMAD2^low^ (CAF subset II)HGF^low^, FGF7^low^, pSMAD2^high^ (CAF subset III)	Hu et al. (2021)
	FAP^positive^ (CAF subset 1)FAP^positive^, αSMA^positive^ (CAF subset 2)FAPTO^positive^, αSMA^positive^, ADH1B^negative^, MYH11^positive^ (CAF subset 3)ADH1B^positive^ (CAF subset 4)	Grout et al. (2022)
Soft tissue sarcomas	Sic2a1, Pgk1, Pkm Pgam,. Hk2, CD73 (glycolytic CAFs)	Broz et al. (2024)

Multiple pathways responsible for tumorigenicity and metastasis are activated by CAFs in cancer cells (see Table 2). Studies have shown that activation of JAK2/STAT3 pathway by CAFs secreting IL6 induces metastasis and invasion in lung, gastric, bladder cancer, and squamous cell carcinoma (Wang et al., 2017; Wu et al., 2017; Goulet et al., 2019; Wang et al., 2019). TGFβ secreted by CAFs activates multiple pathways that sustain a pro-EMT, pro-invasion, and pro-migration phenotype in multiple cancers (Yu et al., 2014; Liu J. et al., 2016; Ren et al., 2018; Yoshimatsu et al., 2020; Ping et al., 2023; Song et al., 2024). In endometrial cancer, activation of PI3K/AKT, MAPK/ERK and SDF1/CXCR4 pathways play a role in proliferation and invasion (Subramaniam et al., 2013; Teng et al., 2016). In hepatocellular carcinoma, CAFs-secreted CXCL11 modulates cell migration and tumor metastasis through the circUBAP2/miR-4756/IFIT1/3 axis (Liu et al., 2021). Hepatocyte growth factor (HGF)-driven activation of PI3K/AKT and ERK pathways plays a role in angiogenesis in gastric cancer and drug resistance in ovarian cancer (Deying et al., 2017; Ding et al., 2018). CAFs-derived IL32 induces breast cancer invasion and metastasis via ITGB3-p38 MAPK signaling (Wen et al., 2019). In colorectal cancer, CAFs promoted stemness, EMT, metastasis, and chemoresistance by secreting exosomes to increase miR-92a-3p and activation of Wnt/β-catenin pathway in cancer cells (Hu et al., 2019). CAFs promote migration and invasion of non-small cell lung cancer (NSCLC) cells via miR-101-3p mediated VEGFA secretion and AKT/eNOS Pathway (Guo et al., 2021). Another study established a CAF-METTL3-RAC3 m^6^A modification-dependent regulation system in NSCLC metastasis (Chen et al., 2023).

**TABLE 2:** Signaling pathways regulated by CAFs in cancer cells.

Signaling pathways	Cancer types and biological processes involved	References
IL-6/JAK2/STAT3	Lung Cancer- Metastasis	Wang et al. (2017)
	Squamous cell cancer-Invasion	Wang et al. (2019)
	Gastric cancer- EMT	Wu et al. (2017)
	Bladder cancer- EMT	Goulet et al. (2019)
TGF-β1/FAP/VCAN axis	Bladder cancer-EMT	Ping et al. (2023)
Chemokine/Hedgehog/TGF-β	Hepatocellular- Metastasis	Liu et al. (2016)
FBXO28 via TGF-beta1/SMAD2/3	Ovarian cancer- Proliferation, migration, and invasion	Song et al. (2024)
TGF-β1/HOTAIR	Breast cancer- EMT and metastasis	Ren et al. (2018)
TNF- α via TGF-β	Oral squamous cell carcinoma- Endo-MT	Yoshimatsu et al. (2020)
TGF-β/Smad	Breast cancer- Enhanced ECM adhesion, migration and invasion, promote EMT	Yu et al. (2014)
CXCL11 via circUBAP2/miR-4756/IFIT1/3 axis	Hepatocellular carcinoma- Cell migration and metastasis	Liu et al. (2021)
PI3K/Akt and MAPK/Erk	Endometrial cancer- Cell proliferation	Subramaniam et al. (2013), Teng et al. (2016)
	Endometrial cancer- Proliferation, migration, and invasion	
SDF-1/CXCR4 axis	Endometrial cancer- Proliferation, migration, and invasion	Teng et al. (2016)
IL32 via integrin β3-p38 MAPK	Breast cancer- Invasion and metastasis	Wen et al. (2019)
Increased miR-92a-3p leading to activation of Wnt/B-catenin pathway	Colorectal cancer- Enhanced stemness and EMT	Hu et al. (2019)
HGF via PI3K/AKT and ERK1/2 signaling	Gastric cancer-Angiogenesis and vascularization	Ding et al. (2018)
HGF via c-Met/P13K/Akt and GRP78 signaling	Ovarian cancer- Cell proliferation and drug resistance	Deying et al. (2017)
METTL3-RAC3 m6A modification	Non-small cell lung carcinoma- Migration and invasion	Chen et al. (2023)
miR-101-3p/VEGFA/AKT	Non-small cell lung carcinoma-Metastasis	Guo et al. (2021)

Overall, the heterogeneity of CAFs coupled with the pro-tumorigenic pathways the CAFs activate in cancer cells suggests that the intimate relationship between the cancer cells and the CAFs creates a feed-forward loop that ultimately facilitates cancer progression, invasion, and metastasis.

## Communication between cancer cells and fibroblasts

Cancer cells are constantly interacting with the components of the TME. Throughout cancer development and metastasis, starting with invasion into the ECM, entering the circulation or lymphatic system, navigating through endothelial linings and basement membranes, and ultimately forming secondary tumors at target sites, effective communication between the cancer cells and the microenvironment is essential at every step (Ungefroren et al., 2011). CAFs, as the major component of the TME, interact with cancer cells either directly, through cell-cell contact, or indirectly via signaling molecules and exosomes within the ECM (Yamaguchi and Sakai, 2015; Liu et al., 2019).

While cancer initiates in the epithelial cells, the presence of carcinoma-associated fibroblasts even before tumor formation can contribute to tumorigenesis (Olumi et al., 1999). However, the latest evidence suggests that CAFs are not tumor-promoting from the get-go. Previous studies showed that early during tumor formation and depending on their activation status, CAFs restrict tumor development (Miyai et al., 2020). This supports a model in which the evolution of cancer cells-CAFs interaction plays a role in generating pro-tumor and pro-metastatic signals, eventually driving cancer progression. Below we summarize the indirect interaction and analyze in detail the role of direct contact between cancer cells and CAFs.

### CAFs-cancer cells indirect interaction

For several cancer types including hepatocellular carcinoma, pancreatic cancer, breast cancer, lung cancer, melanoma, and colon cancer it has been shown that tumor cells interaction with CAFs promotes EMT, metastatic phenotype, and therapy resistance (Kubo et al., 2016; Domen et al., 2021; Shelton et al., 2021; Ahmad Zawawi and Musa, 2022; Ando et al., 2022; Hu et al., 2022). The main indirect mechanisms through which the CAFs influence cancer cells phenotype are: 1) Secretion of collagens, fibronectin, and proteoglycans can remodel the ECM by stretching, crosslinking, bundling, stiffening, and even degrading it, thereby creating a microenvironment more favorable for cancer progression (Scott et al., 2019); 2) paracrine communication through extracellular vesicles (EVs), growth factors and cytokines that promote stemness and metastasis, such as TGFβ, Chemokine (CC motif) ligand 2 (CCL2), IL-6, HGF, Osteopontin (OSPN) and Stromal cell-derived factor 1 (SDF-1) (Linares et al., 2020).

Lately, metabolic reprogramming in cancer cells and the cells in the TME by EVs has been assessed (Chen et al., 2024). EVs are secreted from various cell types and their content comprises of different proteins, microRNAs (miRNAs), DNA, and lipids specific to their parent cells (Castillo-Sanchez et al., 2022). Based on their size, biogenesis, or release pathways, EVs are classified as exosomes, microvesicles, and apoptotic bodies (Castillo-Sanchez et al., 2022). Studies have shown that EVs secreted by CAFs are associated with cancer progression and metastasis in breast cancer, colorectal cancer, and lung cancer (Hu et al., 2019; Kong et al., 2019; Castillo-Sanchez et al., 2022). Kong et al., 2019, demonstrated in their study that in contrast to EVs from salivary adenoid cystic carcinoma (SACC), the EVs from the CAFs facilitated the creation of a pre-metastatic niche favorable for the SACC lung metastasis.

All these mechanisms of indirect communication between cancer cells and CAFs were extensively examined and recognized as critical for cancer progression (Fiori et al., 2019; Liu et al., 2019; Scott et al., 2019; Linares et al., 2020; Hu et al., 2022). While some therapies directed at targeting ECM proteins, ECM remodeling enzymes, and CAF-derived signaling molecules have shown promising results (Tran et al., 2017), others have not (Brandes et al., 2016; Benson et al., 2017), suggesting that targeting the paracrine communication between cancer cells and CAFs is most likely context and case-dependent.

### CAFs-cancer cell direct interaction

**FIGURE 2:**
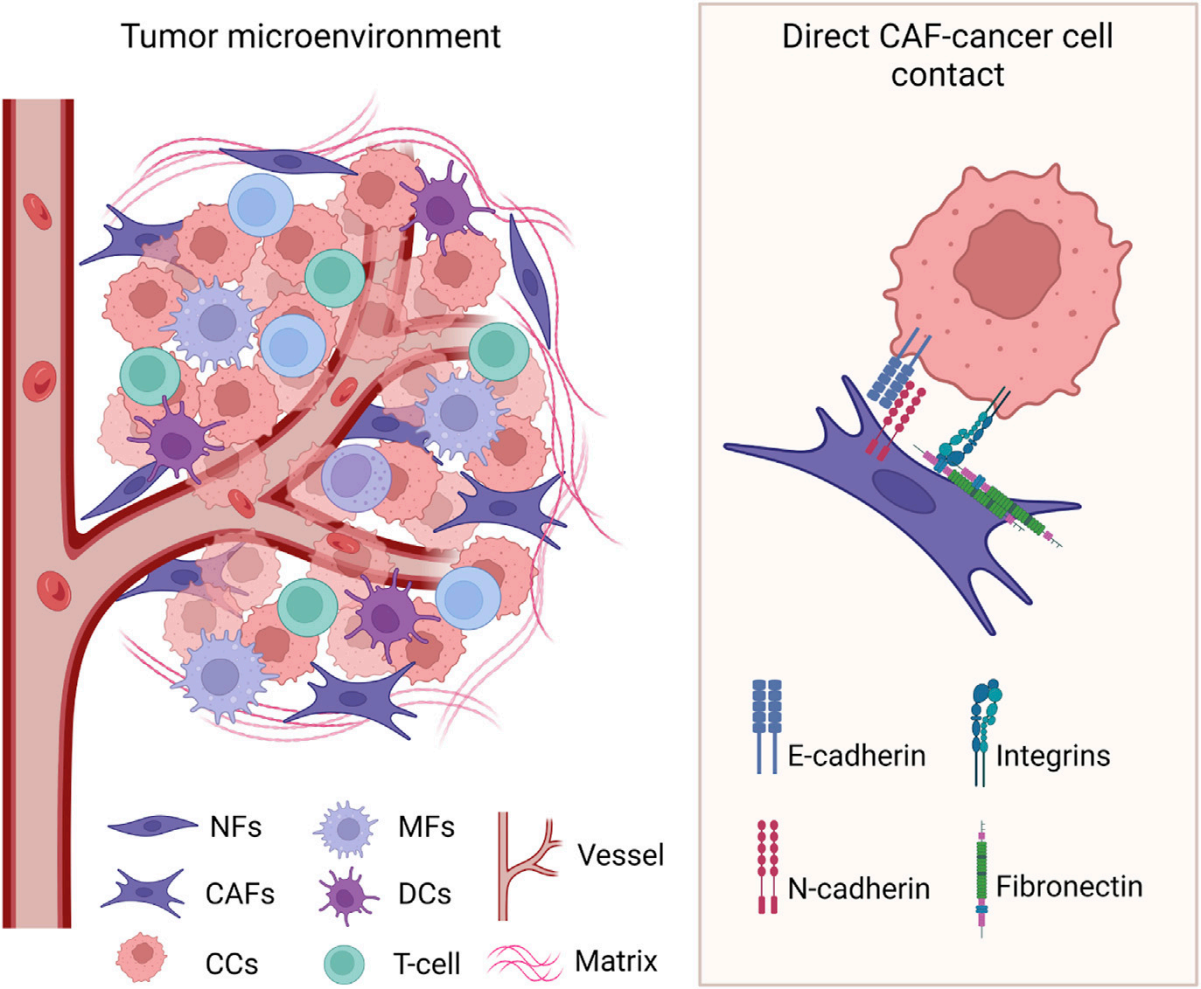
Heterologous direct contact between cancer cells and CAFs in the TME. Known cell surface molecules involved in the interaction are identified. CCs: cancer cells; NFs: normal fibroblasts; CAFs: cancer-associated fibroblasts; MFs: macrophages; DCs: dendritic cells. Created with BioRender.com.

Different from indirect paracrine regulation, direct interaction requires cell-to-cell contact between the cancer cells and the CAFs (Figure 2). Experimental models that use direct co-culture of cancer cells and fibroblasts, as opposed to fibroblast-conditioned media, revealed the multifaceted role of this heterologous direct contact in cancer cells migration, invasion, metastasis, and therapy resistance.

Earlier studies showed that when tumor cells are co-cultured with normal fibroblasts they spread along the fibroblasts and recapitulate the typical *in vivo* tumor morphology (Rønnov-Jessen et al., 1995). In addition, non-activated fibroblasts converted to myofibroblasts in co-culture, supporting a reciprocal cross-talk that sustains tumor growth and invasion. Later, studies using similar co-culture models revealed that squamous carcinoma cells (SCC) require direct contact with CAFs to invade the ECM in a 3D co-culture system (Gaggioli et al., 2007). In contrast, fibroblast-conditioned media or a variety of growth factors were unable to promote the invasion of SCC cells. In this model, the acquisition of motile behavior by SCC cells is not sufficient for invasion, however force-mediated and protease-mediated matrix remodeling is required. These activities are provided by fibroblasts which are the leaders in the invading chains of cancer cells. Further, a reduction in integrins α5 and α3 in fibroblasts dramatically reduces this collective migration by preventing force-mediated matrix remodeling by the fibroblasts (Rønnov-Jessen et al., 1995).

Subsequent studies provided evidence for an impactful role of the direct cell-to-cell contact between cancer cells and fibroblasts in diverse tumor models. For example, conditioned media from breast cancer cells and fibroblasts direct co-cultures increased cancer cells’ metastatic potential while conditioned media from homotypic cultures had little effect, supporting a role for the direct contact between cancer cells and fibroblasts in the release of soluble factors and paracrine signaling (Stuelten et al., 2010). In a prostate cancer co-culture model, fibroblasts promote directional cancer cells migration by organizing the fibronectin matrix (Erdogan et al., 2017). Direct cancer cells-CAFs interaction also enhances the invasion of lung carcinoma cells in a 3D co-culture system (Otomo et al., 2014) and *in vivo* (Duda et al., 2010). Gastric carcinoma studies showed that CAFs promote a strong invasive phenotype when in direct contact with scirrhous gastric carcinoma cells (Yamaguchi et al., 2014; Satoyoshi et al., 2015), whereas CAFs conditioned media did not, emphasizing the importance of direct contact between cancer cells and fibroblasts in invasion (Satoyoshi et al., 2015). Furthermore, TGFβ1 enhances the dissemination of colon carcinoma cells to the liver through attachment to CAFs (Gonzalez-Zubeldia et al., 2015). Activated normal fibroblast cells can attract breast carcinoma MDA-MB-231 cells and function as scaffolds to accelerate aggregation and coalescence of cancer cells (Wessels et al., 2019). This was attributed to the expression of podoplanin (PDPN) by the activated fibroblasts.

To distinguish between the role of normal fibroblasts and CAFs, studies have shown that fibroblasts derived from normal breast tissue inhibit epithelial cells growth, whereas fibroblasts from breast carcinomas have less inhibitory capacity and in turn promote epithelial growth (Sadlonova et al., 2009). Even more, normal fibroblasts grown in direct co-culture contact had a more significant growth suppression effect on cancer cells, indicating that direct contact plays a role in fibroblasts’ role in regulating cancer cells proliferation (Wessels et al., 2019). Further, a zebrafish tumor metastasis model clearly demonstrated that implantation of mixed normal fibroblasts-cancer cells displays little dissemination, while implantation of cancer cells with CAFs increased the number of disseminated cancer cells throughout the zebrafish body (Liu et al., 2017). This is probably due to CAFs enhanced ability to proliferate and potentiate invasion and migration compared with normal fibroblasts in breast cancer, as previously demonstrated (Peng et al., 2013). Altogether, these studies reinforce the idea that a reciprocal signaling system exists between the tumor and stromal fibroblasts and that transformed CAFs in direct contact with cancer cells increase cancer dissemination and metastasis.

The nature of the cell-to-cell contact is critical to understanding how cell surface receptors and signaling molecules involved in the interaction promote activation of cellular pathways driving EMT, cytoskeleton modifications, and resistance to therapy, all signs of cancer reprogramming into an aggressive phenotype. For example, multiple studies showed that integrins are involved in the heterologous cell-to-cell contact between cancer cells and fibroblasts. An earlier study showed that direct interaction with CAFs enhanced pancreatic ductal adenocarcinoma (PDAC) clonogenic growth, self-renewal, and migration (Begum et al., 2019). This was due to EMT induction and an increase in the frequency of CSCs in the population. At the same time, CAFs were activated by PDAC cells and increased collagen synthesis resulting in FAK activation in PDAC cells. Importantly, the knockdown of β1-integrin or the inhibition of FAK kinase activity in PDAC cells abrogated the impact of CAFs on clonogenic growth (Begum et al., 2019). Another study showed that fibroblasts induce contact-dependent migration of colorectal carcinoma cells (Knuchel et al., 2015). In addition, the use of blocking antibodies and integrin antagonists demonstrated that the adhesion between cancer cells and fibroblasts is mediated by the expression of αvβ5 integrin on cancer cells induced through an FGF2-FGFR-SRC pathway. More recently, the direct interaction with CAFs and migration of cancer cells from different tissues (pancreas, lung, colon, and breast) was shown to involve integrin-α5β1 (ITGA5/ITGB1) on cancer cells and fibronectin assembled on the surface of CAFs (Miyazaki et al., 2019; Miyazaki et al., 2020).

Apart from integrins, other cell surface molecules have been identified to play a role in the direct cell-cell contact between cancer cells and CAFs. For instance, a heterotypic and mechanically active E-cadherin/N-cadherin interaction has been described that enables cancer cells invasion (Labernadie et al., 2017). This study demonstrates that the pulling force exerted by the CAFs on cancer cells is mediated by the heterotypic junction between E-cadherin on cancer cells and N-cadherin on CAFs. Importantly, the E-cadherin/N-cadherin interaction is not disrupted by force and creates a leading edge on the opposite side of the cell-cell interaction. Therefore, a cooperative mechanism is formed in which CAFs pull cancer cells away from the tumor and cancer cells polarize CAFs leading edge to further promote their invasion.

Another important aspect of the direct cell-to-cell model is understanding how the interaction is altering the gene signatures on both sides. Fewer studies have dissected transcriptional reprogramming in cancer cells and fibroblasts during and after the interaction. Nonetheless, different transcriptome profiles have been detected in breast cancer cells depending on whether they can directly contact CAFs or not in co-culture models (Camp et al., 2011). In this study, the direct co-cultures appear to have a broader transcriptional response, enriched for more Gene Ontology (GO) functions and more signaling pathways than the transwell indirect co-cultures. Studies focused on understanding the reprogramming in normal fibroblasts after direct contact or “confrontation” revealed that incubation of prostate PC-3 cancer cells in direct contact with fibroblast cell lines induces changes in gene expression patterns in fibroblasts including Rho, the Yes-associated protein 1 (YAP1)/transcriptional coactivator with PDZ-binding motif (TAZ) cascade, pro-inflammatory signaling through NFκB, and TGFβ signaling (Alexeyenko et al., 2015). Further, direct contact of colon cancer cells with bone marrow MSCs, induced CAF-like characteristics in MSCs, such as αSMA expression through activation of Notch and the TGFβ signaling pathway (Zeng et al., 2018).

Recently, we used a small cell lung carcinoma (SCLC) and lung fibroblast *in vitro* model to investigate the effects of direct vs*.* indirect interaction on gene reprogramming in lung cancer cells (Dhungel et al., 2023). We showed that specifically upon direct contact with fibroblasts, cancer cells undergo profound reprogramming and develop a hybrid EMT phenotype in which EMT-inducing growth factors, as well as ECM remodeling proteins, are highly upregulated. In this co-culture model, cancer cells in direct contact with fibroblasts exhibit significantly elevated levels not only of EMT transcription factors (EMT-TFs) but also of other critical EMT markers, including YAP1, FN, TGFβ, CTGF, CYR61, α-SMA, and POSTN. The cancer cells reprogramming by fibroblasts was transient and upon separation from fibroblasts cancer cells slowly reverted to their original more epithelial phenotype. This suggests that in an *in vivo* scenario, the contact with the fibroblasts sustains the hybrid EMT phenotype of the cancer cells, while loss of the contact (possibly during metastasis when cancer cells reach distant sites), would revert the cells to a more epithelial phenotype facilitating secondary tumor formation. Maintaining cancer cells and CAFs direct contact during blood circulation and transition to secondary sites was shown to increase the metastatic potential and viability of lung cancer cells (Duda et al., 2010). The presence of tumor-associated CAFs in brain metastasis from the lung, further suggests that CAFs that accompany cancer cells during metastasis may play a critical role in promoting metastasis and supporting cancer cells survival during transition (Duda et al., 2010). Because the paracrine milieu is distinct under conditions that allow direct contact between cancer cells and fibroblasts and can drive EMT reprogramming in nearby cancer cells (Dhungel et al., 2023), the direct contact could play an initiator role in tumorigenesis at least in certain tissues under specific conditions. The results we obtained from SCLC and lung fibroblasts co-culture are consistent with similar studies using breast cancer cells and normal murine dermal fibroblasts co-cultures (Stuelten et al., 2010). Similar to previous findings, treatment with *ATN-161* (Ac-PHSCN-NH2), a novel small peptide antagonist of integrin α5β1, or depletion of ITGA5 and ITGB1 by siRNA, significantly reduced YAP1 and CYR61 mRNA levels in SCLC H69 cells in direct contact with fibroblasts (Dhungel et al., 2023). This is significant because earlier studies show a specific contribution of ITGA5 and ITGB1-fibronectin interaction in maintaining the survival of growth-arrested cells, potentially by negatively modulating apoptotic response via Akt signaling pathways (Korah et al., 2004).

## Therapeutic implications and challenges

A significant consequence of CAFs interaction with cancer cells, either through direct contact or indirect paracrine communication, is induced therapy resistance. Particularly, ECM produced by CAFs prevents immune cell infiltration into the TME, hampers drug penetration, and diminishes therapeutic efficacy (Gu et al., 2024). Therefore, therapeutic strategies targeting the communication between cancer cells and their TME are actively explored. Previous reviews have described in detail the interplay between CAFs and TME and CAFs and immune cells and their role in chemotherapy resistance (Raskov et al., 2021; Gu et al., 2024; Piwocka et al., 2024). Novel therapeutic approaches include: 1) modulators of gap junctions, 2) integrins blocking antibodies, 3) suppressors of tunnel nanotubes, 4) inhibitors of exosome biogenesis, 5) inhibition of TGFβ and other growth factors cascade, 6) inhibitors of chemokine receptors, and 7) modulators of metabolites (Dominiak et al., 2020).

Most of the newly developed therapies directly targeting CAFs are either in the preclinical or phase I stages (Chhabra and Weeraratna, 2023). These therapies use different mechanisms to either eliminate CAFs (Ko et al., 2016), inhibit the activated state of CAFs (Perera et al., 2017; Palakurthi et al., 2019) or signaling pathways required for CAFs function (Lv et al., 2018; Zhang et al., 2018), and target the CAFs secretome (Sharma et al., 2021; Turaga et al., 2021). One such approach targets the FAP^+^ CAFs in the mouse TME using diphtheria toxin receptor (DTR) expressed selectively in FAP-positive cells and treatment with diphtheria toxin (DTx) (Feig et al., 2013). Because these CAFs also produce immunosuppressive cytokines, depleting this population might be an effective approach to enhance the immune response and alter the ECM production (Feig et al., 2013; Sunami et al., 2021).

Less, however, is known about how the direct contact between the cancer cells and CAFs influences resistance to therapy. One study found that a PDPN-expressing subpopulation of CAFs enhances resistance to epidermal growth factor receptor tyrosine kinase inhibitors (EGFR-TKIs) in lung adenocarcinoma cells during co-culture *in vitro* (Yoshida et al., 2015). Moreover, among patients with lung adenocarcinoma harboring EGFR-activating mutations, cases with the presence of PDPN-expressing CAFs had a relatively poor response to EGFR-TKIs (Yoshida et al., 2015). Resistance to EGFR-TKIs was not observed when cancer cells and PDPN-positive CAFs were cultured in separate chambers, suggesting that direct contact between CAFs and cancer cells is required for the chemoresistance to occur. A follow-up study correlating patient clinical outcomes with the biopsy imaging features confirmed that a higher tumor-tumor interaction is associated with higher benefit from EGFR-TKIs, while a higher tumor-stroma interaction is associated with less benefit from EGFR-TKIs in lung adenocarcinoma (Wang et al., 2023).

Another study aiming to identify inhibitors of the direct contact between CAFs and cancer cells, discovered dasatinib, a Src inhibitor, as a potent blocker of the interaction (Yamaguchi et al., 2014). Importantly, mice treated with dasatinib display less associated stromal CAFs and benefit from an increased therapeutic effect on peritoneal dissemination.

Finally, in a novel study using patient-derived organoids (PDOs) from colorectal cancer and CAFs, the authors were able to re-sensitize CAF-protected PDOs using YAP1 inhibitor Verteporfin (Ramos Zapatero et al., 2023). This is indicative of a role for YAP1 in chemotherapy resistance driven by the direct interaction with CAFs and consistent with observations from our SCLC- lung fibroblasts co-culture model (Dhungel et al., 2023).

Multiple clinical studies aiming to target CAFs and their complex role in tumor progression produced uneven outcomes, probably due to multiple ways of communication between the tumor and the CAFs and yet-to-be-discovered interactions. Although considerable knowledge has been gained about chemotherapy resistance in cancer cells, very little is known about therapy resistance in CAFs or CAFs plasticity under therapy. How direct contact between cancer cells and CAFs changes the phenotypic plasticity and the response to treatment in both cell types should be considered for tailored future therapies.

## Conclusion and future directions

CAFs have emerged as dynamic participants in tumor progression. Their surprising functional diversity associated with specific tissue tumors highlights their critical role in regulating tumor invasion and response to therapy.

The cell-to-cell contact between cancer cells and fibroblasts arises as a critical determinant in EMT, invasion, metastasis, and overall cancer progression. Understanding the intricacies of this communication provides potential targeted therapeutic interventions aimed at disrupting these interactions and, consequently, impeding cancer advancement. This evolving field holds promise for novel strategies in the ever-challenging field of cancer therapy.
